# Neurogenic pectoralis minor syndrome in the differential diagnosis of neck pain: A case series with diagnostic ultrasound-guided pectoralis minor muscle block

**DOI:** 10.1097/MD.0000000000044387

**Published:** 2025-09-12

**Authors:** Kubra Neslihan Kurt Oktay, Feyza Unlu Ozkan, Ilknur Aktas

**Affiliations:** a Department of Physical Medicine and Rehabilitation, University of Health Sciences, Haydarpasa Numune Training and Research Hospital, Istanbul, Turkey; b Department of Physical Medicine and Rehabilitation, University of Health Sciences, Fatih Sultan Mehmet Training and Research Hospital, Istanbul, Turkey.

**Keywords:** brachial plexus compression, neck pain, neurogenic pectoralis minor syndrome, pectoralis minor muscle block, ultrasound-guided injection

## Abstract

**Rationale:**

Neurogenic pectoralis minor syndrome (NPMiS) is an underdiagnosed cause of persistent neck, shoulder, and upper extremity pain. It can mimic cervical radiculopathy or other musculoskeletal disorders, often resulting in misdiagnosis and ineffective treatment. This case series presents 5 patients with NPMiS who achieved symptomatic relief through diagnostic ultrasound-guided pectoralis minor muscle (PMiM) block followed by targeted rehabilitation.

**Patient concerns:**

All patients presented with persistent neck pain often radiating to the scapula or anterior chest wall, along with paresthesia and impaired sleep quality. Prior treatments, including non-steroidal anti-inflammatory drugs, muscle relaxants, and physical therapy, were ineffective. One patient was referred for spine surgery before alternative evaluation.

**Diagnoses:**

The diagnosis of NPMiS was based on detailed clinical history, positive physical exam findings (e.g., Roos test, focal PMiM tenderness), and exclusion of cervical radiculopathy and peripheral neuropathies via imaging and electrophysiological studies. Confirmatory diagnosis was established with significant symptom relief following ultrasound-guided PMiM block.

**Interventions:**

Patients received diagnostic ultrasound-guided injection of 1% lidocaine into the PMiM. This was followed by an individualized rehabilitation program including posture correction, ergonomic adjustments, and physical therapy focused on pectoral muscle stretching and shoulder stabilization.

**Outcomes:**

Patients experienced rapid pain reduction, improved function, reduced medication use, and better sleep. Sustained symptom relief was achieved through adherence to rehabilitation.

**Lessons:**

NPMiS should be considered in the differential diagnosis of chronic, non-dermatomal neck and upper extremity pain, particularly when standard treatments fail. Ultrasound-guided PMiM block is a valuable tool both for confirming diagnosis and initiating effective, personalized therapy strategies.

## 1. Introduction

Thoracic outlet syndrome (TOS) is a condition characterized by compression of upper extremity neurovascular structures from the cervical region to the axilla, leading to pain, paresthesia, weakness, numbness, swelling, coldness, tingling, and discoloration.^[1,2]^ TOS can be classified based on the site of neurovascular compression, which occurs at 3 anatomic levels: the interscalene triangle, costoclavicular space, or pectoralis minor space.^[1,2]^ Pectoralis minor syndrome (PMiS) refers specifically to compression of the brachial plexus nerves, axillary artery, and axillary vein within the pectoralis minor space.^[3]^ Another classification categorizes TOS based on compression location: above the clavicle, where it occurs in the interscalene triangle or costoclavicular space, and below the clavicle, where it occurs in the PM space, leading to PMS.^[2,3]^ The majority of patients experience neurogenic pectoralis minor syndrome (NPMiS) due to brachial plexus compression, while venous PMiS (VPMiS) and arterial PMiS (APMiS) are much less common.^[4]^ The axillary artery and vein, being positioned under the PMiM along the brachial plexus nerves, are relatively resistant to extrinsic compression.^[4]^

Clinical presentations most commonly include pain and paresthesia in the upper extremity, subclavicular anterior chest wall, and axilla, with progressive weakness in chronic cases. Rarely, cyanosis and swelling of the upper extremity may occur.^[4]^ Some patients may also report referred pain in the trapezius, supraclavicular area, shoulder, and occasionally the cervical and upper back regions.^[4,5]^ Physical examination findings primarily include tenderness over the anterior chest wall and axilla.^[5]^ Additional tenderness in the trapezius, rhomboids, biceps, and rotator cuff tendons may also be observed.^[5,6]^

Common causes of PMiS include trauma, repetitive overhead activity, weightlifting, and muscle imbalances caused by poor posture, prolonged sitting, or spasticity, leading to PMiM tightness and shortening.^[7–9]^ Increasing evidence suggests that pectoralis minor compression plays a crucial role in scapular dyskinesia and neurogenic thoracic outlet syndrome (NTOS).^[10,11]^ Differential diagnosis of NPMiS includes carpal tunnel syndrome, cubital tunnel syndrome, pronator teres syndrome, radial tunnel syndrome, cervical spine disorders, shoulder pathologies, Pancoast tumors, and multiple sclerosis.^[12]^ A thorough history and detailed symptom inquiry – including pain distribution, paresthesia, weakness, color changes, coldness, and swelling – combined with physical examination and provocative tests, aid in diagnosis. Imaging modalities such as digital radiography, computed tomography (CT), and magnetic resonance imaging (MRI) can be used to identify structural abnormalities. Electrophysiological studies (EPS), upper extremity venous and arterial Doppler ultrasound, and dynamic venography are essential for evaluating neurovascular compression.^[4,12]^ Ultrasound-guided diagnostic PMiM block using local anesthetic is a key tool for confirming PMiS; a positive block is characterized by immediate relief of tenderness and symptoms.^[6,12,13]^

Management of NPMiS includes both nonoperative and operative approaches. Conservative treatment is the first-line approach, including physical therapy (PMiM stretching, postural correction), manual therapy, non-steroidal anti-inflamatory drugs (NSAIDs), muscle relaxants, analgesics, and adjunctive modalities such as therapeutic ultrasound and transcutaneous electrical stimulation (TENS).^[11]^ For refractory cases, minimally invasive interventions such as dry needling, ultrasound-guided therapeutic PMiM block, and botulinum toxin injections may be applied.^[2,14]^ When symptoms persist despite these interventions, surgical treatment such as pectoralis minor tenotomy (PMT) with partial myomectomy is considered.^[6]^ Recent advancements in ultrasound-guided diagnostic and therapeutic techniques have significantly improved the accuracy of diagnosing and treating PMiS.^[15,16]^

This case series report severe neck pain as a rare presentation of NPMiS. Diagnosis was confirmed through clinical findings and ultrasound-guided PMiM block, leading to successful symptom resolution with conservative treatments. Our findings reinforce the importance of considering NPMiS in the differential diagnosis of persistent neck pain and emphasize the utility of ultrasound-guided diagnostic blocks for accurate diagnosis and targeted treatment.

## 2. Case 1

**Figure 1.**
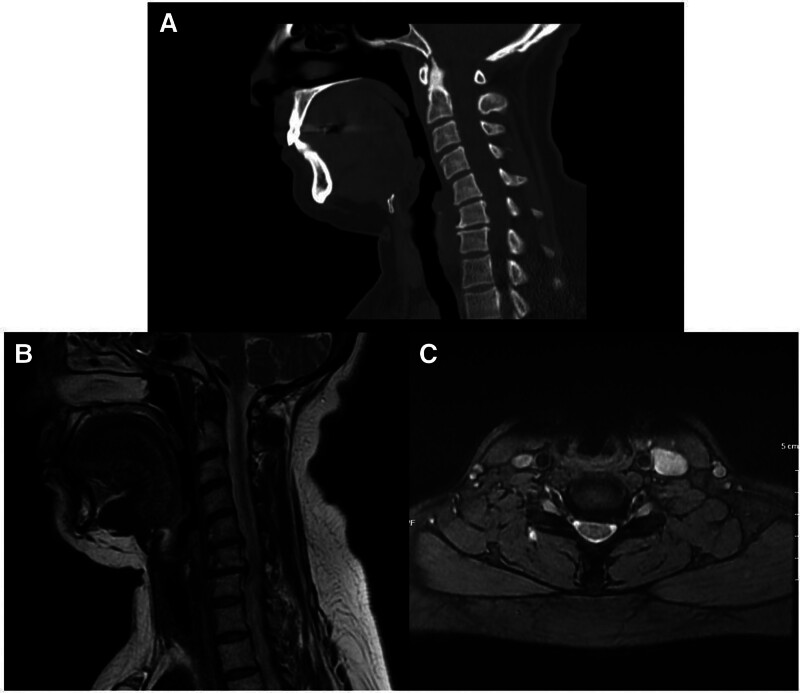
Cervical CT sagittal image showing cervical kyphotic alignment (A), sagittal, (B) and transverse (C) section of cervical MRI of the patient. Broad-based posterior disc protrusion at C5–C6 level, narrowing in anterior subarachnoid space. Left posterior protrusion at C6–C7 disc, compression of the cervical ganglion within the left neural foramen. CT = computed tomography, MRI = magnetic resonance imaging.

A 47-year-old female patient with a prior diagnosis of fibromyalgia was followed at an external center due to severe neck pain, bilateral shoulder and arm pain with paresthesia, inferior scapular pain, fatigue, and weakness. She reported frequent intramuscular muscle relaxant and NSAID injections for pain management. Six months earlier, duloxetine had been prescribed, and she had received a corticosteroid injection in the shoulder joint from an orthopedic surgeon due to persistent shoulder pain. However, severe neck pain persisted, prompting referral to the neurosurgery clinic. CT and MRI revealed cervical angulation (Fig. 1A), a broad-based posterior protrusion at the C5-C6 level, a left-sided posterior protrusion at C6-C7, and compression of the cervical ganglion in the left neural foramen (Fig. 1B and C). EPS identified mild carpal tunnel syndrome in the left extremity, with prolonged distal sensory median nerve latency and reduced sensory nerve conduction velocity, while motor conduction remained normal. Given the correlation between symptoms and root compression, surgery was recommended, but the patient declined and was referred to our outpatient clinic for further evaluation. The clinical symptom distribution, lack of consistent dermatomal findings, and partial or no improvement with cervical-targeted treatments suggested the need for further evaluation of alternative diagnoses. On physical examination, cervical range of motion was painful in all directions, with limited anteflexion. Palpation revealed tenderness in the supraspinatus, infraspinatus, trapezius, and pectoralis muscles, as well as the acromioclavicular joint. Active trigger points were identified in the trapezius, supraspinatus, and pectoralis muscles. The C6 spinous process and paravertebral muscles were tender. Spurling and cervical compression tests were bilaterally positive, as were Neer and Hawkins tests. The Adson test was negative, but ULTT and Roos tests were bilaterally positive. Hypoesthesia was noted in the left C5 dermatome, though motor strength remained intact. Deep tendon reflexes were normoactive bilaterally, with no pathological reflexes. A vascular Doppler ultrasound of the upper extremities was normal. Suspecting PMiS, a diagnostic ultrasound-guided injection into the PMiM was performed, resulting in significant symptom relief, confirming the diagnosis. The patient was informed about the condition and enrolled in follow-up care at our clinic.

## 3. Case 2

A 32-year-old female doctor presented with neck pain, numbness, and tingling in the right upper extremity, particularly in the 4th and 5th fingers. She frequently used NSAIDs and muscle relaxants with minimal relief. Cervical MRI revealed a C5-C6 disc bulge and loss of cervical lordosis. Referred by the neurosurgery department for physical therapy, she underwent EPS, which showed no evidence of cervical root compression, ulnar nerve entrapment, or brachial plexopathy. Upper extremity arterial and venous Doppler ultrasound results were normal. Upon further questioning, the patient reported prolonged sitting – approximately 10 hours daily – while studying for her medical specialty examination. Physical examination revealed painful and restricted cervical range of motion in all directions, while shoulder examination was normal. Palpation elicited tenderness in the trapezius and pectoralis muscles. The Spurling test was negative, whereas the Roos test was bilaterally positive. Motor and sensory examinations were intact, deep tendon reflexes were normoactive bilaterally, and no pathological reflexes were observed. Given her history of prolonged sitting and pectoralis minor muscle tenderness, an ultrasound-guided local anesthetic injection was administered into the right PMiM, resulting in complete resolution of symptoms on that side. One week later, the same procedure was performed on the left PMiM, leading to full resolution of her left-sided symptoms as well. Although the rapid symptom relief supported the clinical suspicion of NPMiS, the diagnosis was based on a multifactorial evaluation including physical examination, history, and lack of evidence for alternative pathologies on imaging and electrophysiological studies. The block was followed by a targeted conservative rehabilitation program, which constituted the primary therapeutic strategy. She was prescribed posture correction and stretching exercises for the pectoralis minor and neck. Additionally, an ergonomic desk chair was provided to optimize her sitting posture. She has remained symptom-free under follow-up for 1 year.

## 4. Case 3

**Figure 2.**
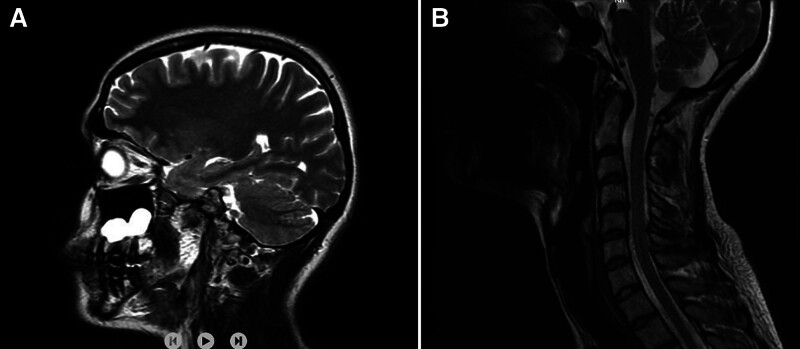
Cranial MRI of the patient (A). Sagittal section of cervical MRI of the patient showing disc bulging at C4–C5 level (B). MRI = magnetic resonance imaging.

**Figure 3.**
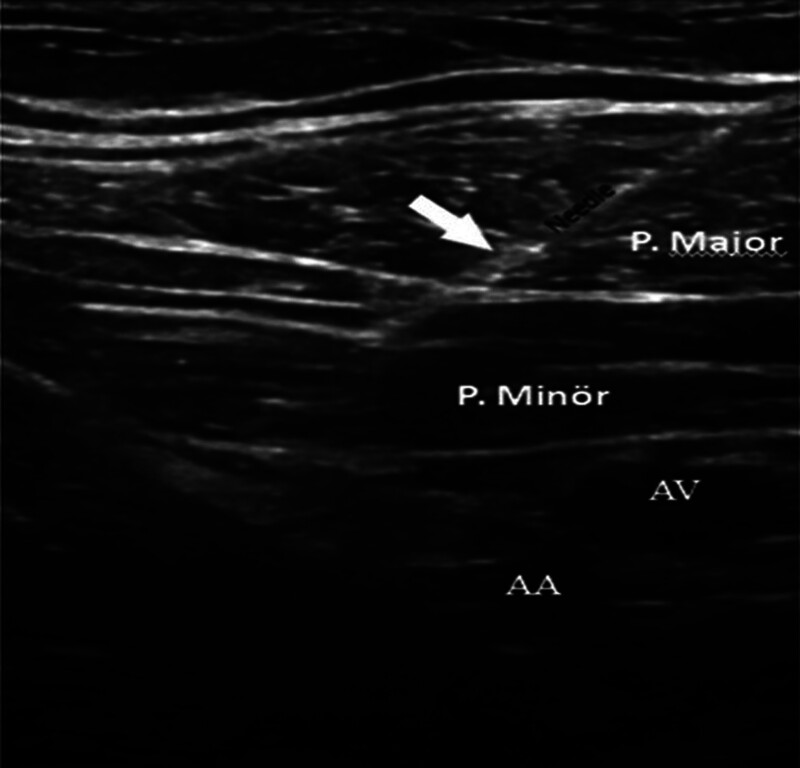
An ultrasound image of pectoralis minor muscle block. Arrow-head indicates the needle. AA = axillary artery, AV = axillary vein, P. Major = pectoralis major muscle, P. Minör = pectoralis minor muscle.

A 25-year-old female patient presented with severe neck pain, occipital pain, trapezius muscle pain, and occasional numbness in both arms and hands, persisting for 2 to 3 years. She had previously consulted neurosurgery and neurology clinics, where cranial and cervical MRIs were performed. The cranial MRI was normal (Fig. 2A), while cervical MRI revealed a large bulging disc at the C4-C5 level (Fig. 2B). Vascular Doppler ultrasound and EPS were normal. Special cervical examination tests provoking radiculopathy including Spurling test were negative, with no sensory or motor deficits. Upon further questioning, the patient reported frequent overhead activities, particularly hanging laundry on a high rod. A detailed physical examination revealed active, painful trigger points in the pectoral muscles, and the Roos test was bilaterally positive. A diagnostic ultrasound-guided injection into both PMiM (Fig. 3) (see Supplemental Video 1 that demonstrates the ultrasound-guided pectoralis minor muscle block technique) resulted in an 80% reduction in neck pain, upper extremity numbness, and PMiM tenderness. The Roos test became negative following the injection, confirming the diagnosis of PMiS. Overhead activities were restricted, and a physical therapy regimen was initiated. This included hot packs, TENS (20 min/d), and therapeutic ultrasound (1.5 W/cm² for 10 minutes) applied to the pectoral and shoulder muscles over 2 weeks. Additionally, a rehabilitation program consisting of gentle pectoral muscle stretching, shoulder stabilization exercises, postural correction exercises, and proprioceptive neuromuscular facilitation stretching for the pectoral and shoulder muscles was implemented 3 times per week for 8 weeks. At the 2-month follow-up, the patient’s symptoms had completely resolved.

## 5. Case 4

**Figure 4.**
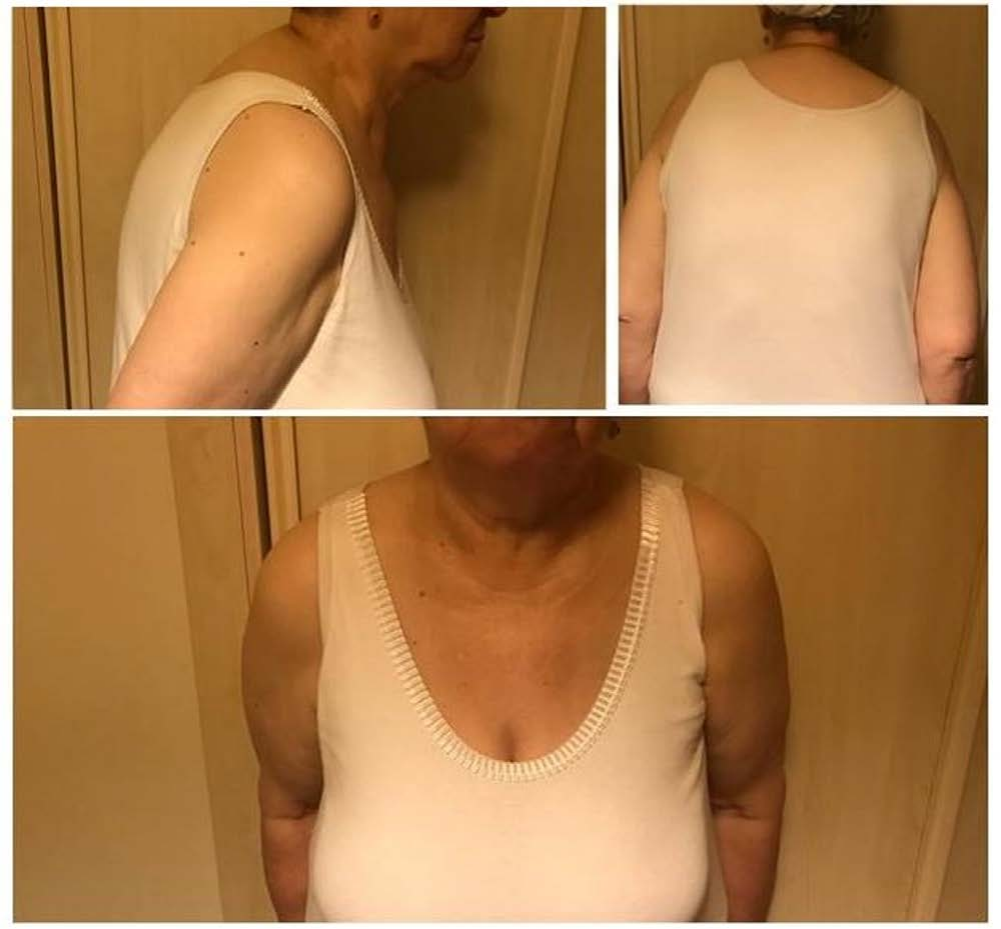
Forward head posture, rounded shoulders, and increased thoracic kyphosis led tight pectoral muscles.

A 71-year-old female patient presented with severe neck pain, particularly at the C7 level, occasionally accompanied by bilateral shoulder pain. Symptoms had worsened over the past 3 months, with the addition of nocturnal pain. NSAIDs and muscle relaxants provided minimal relief. Cervical spine and chest X-rays showed no cervical rib or significant cervical pathology, apart from mild degenerative changes consistent with cervical spondylosis. Laboratory tests and imaging studies, including venous Doppler ultrasound of the upper extremities, were unremarkable. On physical examination, the patient exhibited a forward head posture (FHP), rounded shoulders, and increased thoracic kyphosis (Fig. 4). Point tenderness was noted over the pectoral region. The Spurling test, Lhermitte sign, and Adson test were negative, while the Roos test was bilaterally positive. Provocative maneuvers, such as neck rotation and head tilt, used to assess NPMiS, were mildly positive. Sensory and motor examinations were normal, with no pathological reflexes. Given her postural abnormalities – FHP, kyphosis, and rounded shoulders – contributing to pectoral muscle tightness, a diagnostic ultrasound-guided PMiM block was performed to evaluate for PMiS. Following the injection, the patient experienced complete neck pain relief and reported improved sleep quality.

## 6. Case 5

**Figure 5.**
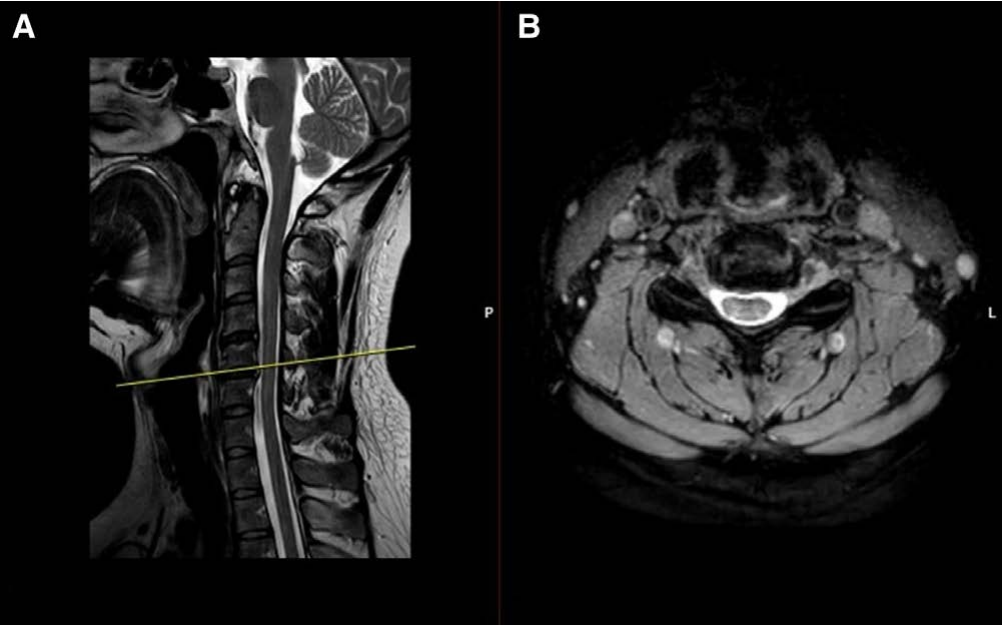
(A) Sagittal section of cervical MRI showing a broad-based posterior central-left paramedian mild protrusion at the C4–C5 level and a broad-based posterior central protrusion at the C5–C6 level. (B) Transverse section of cervical MRI at the C5–C6 level showing narrowing of the right neural foramen due to disc protrusion with ventral root compression, while the left neural foramen remains open with no root compression. MRI = magnetic resonance imaging.

A 31-year-old male patient presented with worsening neck pain. He did not report neuropathic or radicular symptoms. His condition had not improved despite prior medical treatment and physical therapy for cervical disc herniation. Cervical MRI (Fig. 5A and B) revealed a broad-based posterior central-left paramedian mild protrusion at C4-C5 and a broad-based posterior central protrusion at C5-C6, with narrowing of the right neural foramen and ventral root compression. The left neural foramen remained open without root compression. On physical examination, cervical range of motion was minimally restricted and painless. The Spurling test was negative. The trapezius and pectoral muscles were bilaterally tight and short, with tenderness in the left pectoral muscles. No neuromuscular deficits were observed. Laboratory findings were within normal limits. A diagnostic ultrasound-guided left PMiM block was performed, resulting in immediate and significant pain relief. Physical exam findings, lack of radiculopathy, and the immediate symptom relief with PMiM block demonstrated a non-cervical source of the symptoms. The patient was prescribed pectoral and trapezius muscle stretching exercises, along with posture correction exercises. At the 1-week follow-up, his neck and pectoral pain scores had decreased from a VAS score of 8 to 1.

## 7. Discussion

PMiS is characterized by pain, paresthesia, weakness, and/or venous or arterial insufficiency resulting from compression of the axillary neurovascular bundle by PMiM beneath the clavicle.^[1,3,4]^ Compression above the clavicle, occurs in the interscalene triangle and costoclavicular area, producing traditional TOS (interscalene triangle); whereas compression below the clavicle occurs in the PM space, leading to PMiS.^[3,4]^ NPMiS is increasingly recognized as a distinct clinical entity within the spectrum of NTOS. While traditional NTOS primarily involves brachial plexus compression at the interscalene triangle or costoclavicular space, NPMiS results from compression beneath the PMiM in the retropectoralis minor space, producing similar neurogenic symptoms.^[15,16]^

NPMiS is an underrecognized cause of persistent neck pain and upper extremity discomfort.^[15]^ The clinical presentation of NPMiS overlaps with other musculoskeletal and neurological conditions, making accurate diagnosis challenging. Patients may present with neck pain radiating to the shoulder, anterior chest wall, and upper extremity, often accompanied by paresthesia and weakness in advanced cases.^[14]^ Due to symptom similarities, NPMiS is frequently misdiagnosed as cervical radiculopathy, myofascial pain syndrome, or other neurovascular entrapment syndromes.^[4,17]^ Tenderness localized to the pectoralis minor muscle and anterior chest wall, often absent in other neurovascular entrapment syndromes, serves as a distinguishing feature.^[18]^ Tenderness over the pectoralis minor muscle and exacerbation with scapular protraction or overhead activity are key physical exam findings.^[16]^ Additionally, brachial plexus provocative tests such as Adson or Roos may be negative, further complicating the diagnosis.^[19]^

Differentiating NPMiS from other neurovascular compression syndromes in the same anatomical region is essential for appropriate management. While traditional NTOS typically presents with ulnar-sided pain, paresthesia, occipital headache, and tenderness in the supraclavicular region and scalene muscles, NPMiS more often manifests with shoulder pain, anterior chest wall tenderness, and axillary discomfort.^[4,17,20]^ Symptoms in NPMiS are predominantly localized to the hand, arm, and chest, with minimal neck involvement, unlike traditional NTOS, which tends to include more prominent head and neck complaints.^[20]^ Palpable tenderness over the PMiM, anterior chest, and axilla is more characteristic of NPMiS.^[18]^ Furthermore, provocative tests such as Adson, Roos, and Wright – frequently positive in NTOS – may be negative in NPMiS, supporting the utility of PMiM block as a diagnostic aid.^[19]^

All 5 cases in this study presented with severe neck pain, a relatively uncommon but clinically relevant manifestation of NPMiS. As the site of compression lies below the clavicle, NPMiS is often overlooked in patients with persistent neck and upper extremity symptoms.^[13,14]^ A thorough history and physical examination are essential to distinguish between supraclavicular and infraclavicular brachial plexus compression. In the absence of trauma, clinicians should inquire about occupational and recreational activities, particularly those involving repetitive overhead arm use, which may stress the PMiM due to its attachment to the coracoid process. Sports such as swimming, baseball, volleyball, and weightlifting are recognized risk factors.^[1,6]^ In Case 3, frequent overhead arm use while hanging laundry contributed to PMiM trigger point formation and NPMiS.

Beyond mechanical nerve compression, PMiM shortening due to muscle imbalance, poor posture, prolonged static positioning, and repetitive overhead movements are critical contributors to the development and persistence of NPMiS.^[7–9,21]^ FHP and scapular dyskinesia increase chronic tension in the PMiM, heightening neurovascular compression risk.^[13,14]^ Poor posture patterns, including upper crossed syndrome – characterized by tightness in the upper trapezius, levator scapulae, and pectoral muscles, coupled with weakness in deep cervical flexors and scapular stabilizers – are frequently observed.^[7,8]^ In Case 4, FHP, rounded shoulders, and thoracic kyphosis led to PMiM tightness. Similarly, in Case 2, prolonged sitting and postural imbalance played a key role in symptom onset.^[1,7]^ A study has shown that latent trigger points in the PMiM may worsen rounded shoulder posture, perpetuating muscle tightness.^[22]^ These postural abnormalities are also prevalent in athletes, particularly swimmers, and increase susceptibility to NPMiS.^[7,8,21]^

Given the broad differential, a comprehensive history and focused physical examination are essential for distinguishing NPMiS from other neurovascular compression syndromes. Electrophysiological studies are frequently normal in NPMiS and are primarily employed to exclude peripheral neuropathies and cervical radiculopathies.^[6]^ While MRI and CT scans can help rule out spinal or shoulder pathology, they do not confirm PMiS directly.^[3,5]^ Vascular forms of TOS, such as subclavian vein or axillary artery compression, may present with overlapping symptoms, and dynamic Doppler ultrasound is valuable in evaluating such cases.^[4,5,13]^

The ultrasound-guided PMiM block remains the most reliable key diagnostic clinical test, providing rapid and localized symptom relief indicative of NPMiS.^[14]^ It is a safe, cost-effective, and practical technique that allows precise targeting of the pectoralis minor while avoiding neurovascular structures. Typically, 4 cc of 1% lidocaine is injected at a 45° angle, with symptom improvement observed within 30 to 40 minutes.^[1,2,4,13]^ In our series, all patients showed substantial pain relief following PMiM block, underscoring its diagnostic utility. For instance, Case 1 was initially recommended for cervical spine surgery, but PMiM block confirmed NPMiS and spared the patient unnecessary intervention.

Although cervical discogenic or radicular sources are often considered first in patients with neck and shoulder pain, several anatomical and clinical studies demonstrate that compression of the brachial plexus cords by the pectoralis minor muscle – particularly the medial and lateral cords – can produce referred pain not only to the anterior shoulder and upper chest but also to the lower neck region, especially under dynamic or postural strain. This mechanism has been emphasized in the literature on nNTOS and PMiS, highlighting its ability to mimic cervical pathology even in patients with normal EMG findings and non-correlating imaging.^[1,3,23]^ In our series, the absence of dermatomal correlation, normal EMG results, and poor response to standard cervical interventions supported the diagnosis of NPMS as the dominant etiology. These findings underscore the importance of comprehensive clinical assessment beyond imaging alone.^[24]^

Although the pharmacological effect of short-acting local anesthetics typically lasts only a few hours, their downstream biomechanical and neuromuscular consequences – such as muscle relaxation, improved scapular mechanics, and reduced peripheral sensitization – can lead to symptom relief lasting days or even weeks in selected patients.^[2,25]^ This observation aligns with prior clinical reports and systematic reviews of dynamic muscular compression syndromes, where temporary muscle inactivation appears to reset maladaptive neuromuscular tension patterns.^[25,26]^

In our case series, the ultrasound-guided PMiM block was not used as a stand-alone diagnostic test but rather as a supportive component within a multifactorial framework. We acknowledge the potential for confirmation bias, but all patients underwent comprehensive evaluations – including detailed clinical history, absence of dermatomal distribution, normal EMG findings, lack of response to cervical interventions, and positive findings specific to PMiS – before diagnosis was established.

Importantly, the prolonged symptom relief observed in several cases was not solely attributed to the block itself. Instead, the block acted as a diagnostic tool that enabled the initiation of targeted therapeutic strategies such as postural correction, ergonomic modifications, and a structured rehabilitation program focused on pectoralis minor stretching and scapular stabilization. Patient adherence to these interventions played a central role in long-term symptom control.^[11,14,16]^ Exercise-based rehabilitation – including stretching, strengthening, postural reeducation, and ergonomic adjustments – has been shown to significantly improve symptoms and function in patients with neurogenic TOS and PMiS.^[27–31]^ These multimodal programs address underlying anatomical compression and help restore scapulothoracic mechanics, supporting long-term recovery. Accordingly, in our series, sustained relief was primarily attributed to adherence to these rehabilitation strategies rather than to the diagnostic block alone. Thus, we believe that prolonged relief is more plausibly achieved through a multimodal therapeutic response.

Nonetheless, we recognize the absence of long-term follow-up in all cases as a limitation of this report. In clinical practice, recurrence of symptoms after initial improvement may warrant escalation to ultrasound-guided corticosteroid injection, which can offer prolonged anti-inflammatory and neuromodulatory benefits.^[^^2]^ In more refractory cases, botulinum toxin injections into the PMiM have demonstrated efficacy in reducing muscular tension and neurogenic symptoms.^[^^2,4,17,32^^]^ In refractory cases, surgical intervention, PMT, has been shown to provide significant symptom relief with minimal morbidity.^[^^19,32,33^^]^

Although placebo-controlled or comparative blocks are considered the gold standard in interventional diagnosis, they were beyond the scope of this case series and not feasible in our observational design. Instead, our diagnostic approach was multimodal and pragmatic, incorporating clinical history, physical examination findings specific to PMiS (e.g., focal tenderness, provocative test responses), lack of dermatomal correlation, poor response to prior cervical interventions, and normal electrophysiological findings. This comprehensive framework allowed for a real-world diagnostic reasoning process in line with previous reports and expert recommendations.^[25,26,29]^ The ultrasound-guided PMiM block was thus used not as a definitive diagnostic test, but as a supportive tool to strengthen the clinical impression and guide further therapeutic planning.

To our knowledge, no previous case series has reported patients presenting with isolated neck pain as the predominant symptom of underlying PMiS. A thorough clinical history, targeted physical examination, and ultrasound-guided diagnostic muscle block remain the cornerstones of accurate diagnosis. Our findings underscore the importance of considering NPMiS in the differential diagnosis of persistent, treatment-resistant neck pain and demonstrate the utility of this approach in confirming NPMiS and guiding effective treatment strategies.

## 8. Conclusion

NPMiS is an underrecognized and often misdiagnosed cause of persistent non-dermatomal, treatment-resistant neck and upper extremity pain, frequently mimicking cervical radiculopathy or other neurovascular compression syndromes – potentially leading to misdiagnosis and unnecessary interventions. Accurate diagnosis requires a detailed clinical history, targeted physical examination, and the use of ultrasound-guided PMiM block as a safe and practical adjunctive tool. In this case series, all patients exhibited substantial symptomatic relief following PMiM block, which not only supported the diagnosis but also enabled the initiation of effective, personalized rehabilitation strategies.

## Acknowledgments

The authors would like to thank the patients who participated in this case report for their trust and cooperation.

## Author contributions

**Conceptualization:** Kubra Neslihan Kurt Oktay.

**Data curation:** Feyza Unlu Ozkan, Ilknur Aktas, Kubra Neslihan Kurt Oktay.

**Formal analysis:** Feyza Unlu Ozkan, Kubra Neslihan Kurt Oktay.

**Investigation:** Feyza Unlu Ozkan.

**Project administration:** Ilknur Aktas.

**Supervision:** Ilknur Aktas.

**Visualization:** Ilknur Aktas.

**Writing – original draft:** Kubra Neslihan Kurt Oktay.

**Writing – review & editing:** Kubra Neslihan Kurt Oktay.
